# The Prevalence, Cause, and Treatment of Bovine Abortion, Milk-fever, and Garget

**Published:** 1898-03

**Authors:** 


### REVIEW.

THE PREVALENCE, CAUSE, AND TREATMENT OF BOVINE ABORTION, MILK-FEVER, AND GARGET. By JULIUS NELSON, Biologist. November 30, 1897. Bulletin No. 127, New Jersey Agricultural Experiment Stations.

NEW JERSEY is entitled to the credit for a great discovery. It has been supposed for a number of years that our immense agricultural and live-stock interests require experiment stations, where experts could be employed to make thorough studies of various subjects connected with these interests, so that farmers might be placed in a position to avoid injurious influences, to encourage favorable influences, to adopt new methods from time to time, and thus receive a greater reward for their labors. Acting upon this assumption, the Congress of the United States has appropriated enormous sums for the establishment and maintenance of at least one such station in every State, and experts of various kinds have been employed to conduct experiments and publish their reports, all for the good of the struggling farmer. Some, indeed, have gone so far as to maintain that this work has already produced good results, and that it should not only be continued, but that it should be enlarged and strengthened.

Some recent publications, however, have shown that these generally accepted views are wrong; that such experiment stations are not necessary, and that the work that they are doing can be accomplished at much less expense, permitting a saving of several hundred thousand dollars each year. Fields, farms, laboratories are all unnecessary; the money spent for special buildings and equipments, especially in the veterinary divisions of experimental work, has been wasted. If we may judge the work of these stations by publications similar to the one whose title appears above, it were better to sell to the highest bidder the various experiment-station equipments of the country, invest the proceeds in Government bonds, rent a few rooms in some convenient office-building near a large library, employ a few bright young men to collate facts that have been recorded, to interview farmers by mail, arrange articles on various subjects relating to agriculture, and publish these results in bulletin-form and distribute them after the manner of a free newspaper. It is not necessary that such employés should have any great amount of knowledge of the subject that they write about. They are supposed to consult the writings of experts, and original views will, therefore, be unnecessary.

There is very little to say about this bulletin except that it collates a number of facts in reference to these diseases that have frequently appeared in text-books and in the journals of the profession. Although the title might lead one to think that abortion, milk-fever, and garget were all produced by the same cause, and yield to the same treatment, this deduction is not borne out by an examination of the text. Prof. Bang's work on the *Etiology of Abortion* has been generously drawn upon, and his deductions are quoted, in the language of the author of the bulletin, at considerable length.

Among the original plans of treatment, it is mentioned that a teaspoonful of sulphur was given once each week to every cow in the herd; but apparently this did not prevent the occurrence of abortion.

The internal treatment for garget is interesting. It consists, if the cow has fever, in administering one ounce of saltpetre and fifteen drops of aconite in one pint of water every four hours. "A wet-pack may be indicated in rare instances, but it should always be used with great care." How many farmers know what a wet-pack is, what rare instances require it, or what care should be observed in its use? Although five kinds of garget are mentioned, all of which require entirely different treatment, the herdsman is not informed how to distinguish the different varieties, or what treatment each requires. Tubercular garget is mentioned by name only; nothing is said as to its symptoms, nor is attention called to the extreme danger accompanying the use of milk in these cases. In connection with the treatment of garget, we note the following: "After all the milk is drawn a 1 per cent. creolin solution, or 1 : 1000 corrosive sublimate solution, may be injected into the teat and forced up until the gland has received all that it will receive. Then milk out"! We hope that this treatment will not come into general use in New Jersey.

As to the treatment of milk-fever, it is rightly stated that "an ounce of prevention is worth forty pounds of effort to cure," although it is further stated that seventeen cow-owners have recorded their methods of treatment, which have proved eminently successful. "Should the owner fear that his method has not succeeded in reducing the blood sufficiently, as may happen when the time is too short, a veterinarian should be called to let out several quarts of blood from the jugular vein of the neck (*sic*)." This advice is very good, and will tend to greatly lessen the trials of the New Jersey veterinarians, because instead of having the responsibility of cases of milk-fever upon their own shoulders, it will only be necessary for them, hereafter, to carry out the plan of treatment directed by the farmer. "Neither carbolic acid nor corrosive sublimate nor iodoform can be recommended for disinfecting the genital passages, but these disinfectants and others may be used liberally upon the soiled bedding, barn-floor, etc." Would not a few pounds of iodoform make an admirable stable disinfectant? "If the cow is down, she should be propped up on the brisket to prevent the running of food into the lungs from the stomach"! Further comment is unnecessary.

